# Local field potential phase and spike timing convey information about different visual features in primary visual cortex

**DOI:** 10.1186/1471-2202-12-S1-P248

**Published:** 2011-07-18

**Authors:** Alberto Mazzoni, Christoph Kayser, Yusuke Murayama, Juan Martinez, Rodrigo Quian Quiroga, Nikos K Logothetis, Stefano Panzeri

**Affiliations:** 1 RBCS Department, Italian Institute of Technology, Genova, 16163, Italy; 2 Max Planck Institute for Biological Cybernetics, Tuebingen, 72076, Germany; 3 Department of Engineering, University of Leicester, Leicester. LE1 7RH, UK; 4 Faculty of Life Sciences, University of Manchester, Manchester. M601QD, UK

## 

The natural visual environment is characterized by both “what/where” aspects (image features such as contrast or orientation which are defined by the relationship between visual signals simultaneously presented at different points in space) and “when” aspects, describing the temporal variations of the image features. Both “when” and “what/where” information is necessary to describe and understand the natural visual environment, and to take appropriate behavioral decisions. While “where” can be considered embedded as retinotopy, it is likely that localized neural populations in the visual cortex keep a simultaneous representation of both “what” and “when” aspects of the visual stimuli. However, little is yet known about how the spike trains of neurons in primary visual cortex encode both sources of information.

The traditional hypothesis in systems neuroscience is that sensory variables are represented by a rate code, i.e. all sensory information is encoded by the number of spikes emitted over relatively long time windows. Although the relevance of rate in encoding static features is well established, this code can be inherently ambiguous in changing environments [1] and it is unlikely that this code is rich enough to represent simultaneously different types of information. Therefore here we explore the hypothesis that the timing of spikes is a crucial variable in representing both “what” and “when” aspects of the natural visual environment.

To address these issues, we recorded single unit activity and LFPs in primary visual cortex of opiate anaesthetized macaques during the binocular presentation of naturalistic color movies. By means of computational analysis, we extracted several image features (color, orientation, luminance, space and time contrast, motion) from the receptive fields of each single neuron. We then considered two different spike timing codes previously studied in both the auditory [2] and the visual cortex [3]. In the first code, which we call spike patterns code, sequences of spike times from single neurons are measured (with a resolution of the order of 10 ms) with respect to the time course of the external stimulus. In the second code, which we call phase of firing code, spikes are measured with respect to the phase of the concurrent low frequency LFPs recorded from the same electrode as the spikes. We then used these data to investigate systematically which types of neural codes carry information about the static features of the image and which neural codes carry information about the time course of these features. We found that both “when” and “what” aspects are encoded simultaneously by spike times of visual cortical neurons. However, “what” and “when” are encoded by two different neural information streams; “what” aspects are encoded (on a fine scale of few ms) by spike patterns, and “when” stimulus aspects are encoded by the phase of firing (on a coarse scale of hundreds of ms).
